# On the Pharmaceutical and Toxicological Applications of Carbon

**Published:** 1850-01

**Authors:** 


*On the Pharmaceutical and Toxicological Applications of Carbon.* By M. Esprit.—M. Esprit believes that the property possessed by carbon of appropriating saline matters held in solution, is not sufficiently known. Payen, twenty-five years since, announced that such is its separating power in respect to lime and the calcareous salts, that if 100 parts of distilled water, saturated with lime, be boiled with 10 parts of charcoal, the filtered liquor will show no traces of the lime. Some years later, this was shown to apply, to a certain degree, to the alkalies. In 1829, Graham studied the action of carbon on nitrate of lead, arsenious acid, nitrate of silver, sulphate of copper, sulphate of ammoniated copper, the hydrate of lead dissolved in potass, solution of iodine, and the chlorides of soda and lime. He was unable to precipitate the arsenious acid, or sulphate of copper, but succeeded with all the others. Dupasquier showed that it energetically absorbs a large proportion of the alkaline sulphurets. These facts had made but little impression, when Chevalier announced in 1845, that the acetate and nitrate of lead, held in solution in water, wine or vinegar, are removable by carbon, with or without the aid of heat. Garrod having thrown some doubts upon Graham's experiments, M. Esprit resolved to repeat and extend them, and now communicates the results at which he has arrived. He found that the following salts were energetically absorbed: the acetate and sulphate of copper, the acetate, sulphate, and chlorate of zinc, oxide of zinc dissolved in potass, the acetate and nitrate of lead, acetate and nitrate of iron, tartar emetic, nitrate and sulphate of silver, chloride of silver in ammonia, corrosive sublimate, nitrate of cobalt, sulphate of cadmium, and the chloride of barium. He found that the sulphate of soda, potass, and magnesia, arsenious acid, and the nitrate of copper, were very sparingly separable. Five parts of the carbon of the blood, calcined with potass, and washed, produced such complete precipitation of the following salts, that no trace could be found in the filtered liquor, viz: sulphate of ammoniated copper, acet. and nitr. of lead, sulph. and nitr. of silver, chlor. of silver in ammonia, chlor. of zinc, and oxide of zinc in potass. Twenty parts were required for the following, viz:

sulph. and acet. copper, corros. subl., tart. emet., nitr. cobalt, sulph. zinc, sulph. cadmium, and chlor. barium.

M. Esprit's experiments agree with those of Mandrel and Bussy, in denying the great absorbability of arsenious acid declared by Garrod to occur; and so small is the proportion separated, that the utility of carbon as an antidote in poisoning from arsenic may be considered as *nil.* The general conclusions are the following:

1. Carbon, as regards prescribing, is incompatible with the substances which it appropriates and renders insoluble. 2. Its use in chemico-legal researches may give rise to great errors; for, as Chevalier has remarked, authors frequently direct us to discharge, by means of carbon, the colour of liquids, in which we have afterwards to seek for the presence of metallic salts removable by carbon. Thus, even in modern works, we are told to decolorize, by its use, liquids in which we have to declare whether a salt of lead, &c., is present! 3. In some cases, the property which carbon has of fixing certain bodies round its particles much facilitates their detection; thus, for the separation of corrosive sublimate, and, as shown by M. Lebourdais, of vegetable alkalies, it suffices, after treating the liquids containing them with carbon, to remove them from this body by alcohol or ether. 4. If a better is not at hand, charcoal may be given as an *antidote* against all the poisons it thus has the power of fixing. For this end it should be given, after vomiting, in large quantities of fine powder suspended in water.—*Brit. and For. Med. Chir. Rev., from Bull. de Therap.*

